# How silanization influences aggregation and moisture sorption behaviours of silanized silica: analysis of porosity and multilayer moisture adsorption

**DOI:** 10.1098/rsos.180206

**Published:** 2018-06-06

**Authors:** Jun Jiang, Jinzhen Cao, Wang Wang, Jing Xue

**Affiliations:** MOE Key Laboratory of Wooden Material Science and Application, Beijing Forestry University, Haidian, Beijing 100083, People's Republic of China

**Keywords:** silanized silica particles, agglomerates, nitrogen adsorption, moisture adsorption, Hailwood–Horrobin model, adsorption hysteresis

## Abstract

Based on the results of nitrogen adsorption and dynamic vapour sorption as well as analysis by the Hailwood–Horrobin (H-H) model, the effects of γ-methacryloxypropyltrimethoxysilane (MPTS) on the agglomeration and moisture sorption properties of fumed silica particles were investigated. After adding various concentrations (2%, 4%, 6% and 8%) of MPTS, different degrees of silanization were obtained by showing various ─OH group contents on the silica surface, which resulted in silica agglomerates with different porous structures. The bigger mesopores in the unmodified silica agglomerates became smaller and finally disappeared after MPTS modification and the Bruanuer–Emmett–Teller surface area decreased more gradually with an increase in MPTS concentration. The H-H model fitted the sorption isotherms very well, and both hydrated water and dissolved water showed decreasing trends with the increase in MPTS concentration, showing reduced hygroscopicity. Up to 6% MPTS, the ─OH groups decreased with increasing MPTS concentration, as indicated by reduced *K*_h_ and *W* parameters, while at 8% MPTS an extensive self-condensation of MPTS occurred. Adsorption hysteresis appeared for moisture sorption on silanized silica, especially at low relative humidity values and at low MPTS concentrations, which could be explained by a synergistic effect of the surface ─OH group content and pore characteristics. These results could aid our understanding of the applications of silane-modified silica particles.

## 1. Introduction

Silica particles have received considerable attention because of their special physical and chemical properties. They can be used as additives, polishing materials, pigments, free-flow agents (in particles), medicinal and industrial adsorbents, [1–3], and in any other unconventional fields. For example, they can serve as a solid emulsifier in Pickering emulsions, which generally require amphipathicity of the silica surface [4,5]. However, plenty of spectral and chemical data have unambiguously substantiated the presence of hydroxyl groups (─OH) on the surface of silica particles [6–8], which result in agglomeration between silica particles and also provide a reaction site for chemical design. As a result, the silica surface is generally hydrophilic and easy to agglomerate, and it has negative effects on compatibility and emulsion stability when used with an organic polymer matrix and as an emulsifier, respectively, which greatly restricts the application of silica. Therefore, the silanization of the silica surface is very important in practical surface chemistry, especially in chemical, pharmaceutical and particle-stabilized emulsions (Pickering emulsions). The degree of silica surface silanization can influence the surface hydrophilicity as well as the final product design, production processes and properties [9–11].

In addition, with regard to porous adsorbents, a wide range of materials have been reported, including silica, porous carbons and metallo-organic frameworks [12–14]. Among these, porous silica is one of the most frequently used water or gas adsorbents and has gained significant attention in the scientific community, especially silica functionalized with different groups [15,16]. This is mainly due to its low production costs and widely adaptable temperature range. There have been some reports revealing the effect of silanization on the pore characteristics of porous silica [17,18]. However, for fumed silica particles (non-porous), the loose agglomeration creates inter-particle voids, which can be treated as ‘pores’ in some instances. Understanding the characteristics of these pores and the water adsorption properties of agglomerated silica is also of great importance, especially for silica with different degrees of silanization. Ridaoui *et al*. [19] studied the interaction between water and fumed silica modified by a controlled partial silylation with dimethyldichlorosilane and found the high grafting ratio exhibited a high temperature dependence on immersion heat. Khalfi *et al*. [20] used inverse gas chromatography (IGC) to investigate interactions between silica and poly(dimethylsioxane) (PDMS) and found that PDMS oligomers are very good at interacting with silylated silica, that is, PDMS oligomers can be used as powerful IGC probes to study the capacity of interaction of a silylated silica with a PDMS elastomer. However, few studies have focused on the ‘pore' structure between fumed silica particles with partial silanization and its effect on moisture adsorption. Moreover, this can be viewed as an approach to characterize the effect of silanization on the agglomerated behaviour of fumed silica particles. Also, due to the hydrophobicity of silanes, water vapour sorption on fumed silica with different degrees of silanization as well as its relation to ‘porosity’ is another characteristic that should be investigated.

For silanization, γ-methacryloxypropyltrimethoxysilane (MPTS) is a widely used coupling agent because of its dual-functional groups. It can be hydrolysed in aqueous solution and the equilibrium between hydrolysis and condensation can be adjusted by changing the hydrolysis conditions such as the hydrolysis time [21]. Li *et al*. [22] used MPTS and octylphenol polyoxyethylene ether (7) (OP-7) to modify SiC/SiO_2_ powder and found a significant improvement in hydrophobicity. Krysztafkiewicz *et al*. [23] investigated the effect of silane coupling agents, including MPTS, on the dispersion properties of sodium–aluminium silicates. Their study showed that the methacryloxy in MPTS could react with methyl methacrylate (basic components of dispersion paints), which improved the sedimentation behaviour in potassium metasilicate. Generally, the modified process by MPTS can be accomplished by the chemical reaction between the trimethoxy groups of silane molecules and the ─OH groups on the silica surface, whereas the other functional group of the silane molecule may remain unchanged [24,25].

This study provides an insight into the porous properties of the surface of silanized fumed silica agglomerates modified by different concentrations of MPTS. The silanol content and moisture adsorption behaviour were characterized. In addition, the influence of MPTS on the characterization of pore size distribution, pore volume and the specific surface area were also determined by nitrogen adsorption experiments, which can reflect the effect of silanization on silica agglomeration. The moisture adsorption behaviour was tested by the dynamic vapour sorption (DVS) approach, which was able to provide accurate isotherms over a wide relative humidity (RH) range and temperature. This method was used to investigate the sorption properties of hygroscopic materials, such as natural fibres and food powders [26–28]. Based on these, the Hailwood–Horrobin (H-H) model, which was used to analyse the moisture sorption behaviour of hydrophilic materials such as wood, food and textiles, was applied to further analyse the moisture adsorption characteristics of silica particles. The findings of this study aim to contribute to an improved understanding of fumed silica agglomerates with various degrees of silanization and the reactive adsorption of water on these agglomerates, which could provide guidance for the design of silica surface modifications for application in desiccants or functional modifiers.

## 2. Experimental

### 2.1. Materials

Hydrophilic fumed silica particles were purchased from Degussa AG (Frankfurt, Germany) in powder form. The Bruanuer–Emmett–Teller (BET) surface, as determined by the manufacturer, was 220–300 m^2^ g^−1^, and the particles’ size was 30–40 nm. The water dispersion of silica was characterized at 25°C by a laser particle analyser (Delsa Nano C; Beckman Coulter, USA), and the average particle size was found to be 350 nm. The difference between the diameter of the primary hydrophilic silica particles, as provided by the manufacturer, and their measured average diameter after dispersion in aqueous solutions could be ascribed to the agglomeration of the particles inherently taking place in dry nanoparticles [29]. MPTS (purity ≥ 95%) was purchased from the market with a density of 1.04 g cm^−3^. Ethyl alcohol, as the diluting agent, was an analytical reagent and was purchased from Beijing Chemical Co., Ltd. of China. The pH values were determined automatically (FE20; Mettler Toledo, Switzerland).

### 2.2. Preparation of variously silanized silica particles

The MPTS at different concentrations (2%, 4%, 6% and 8% based on silica weight) was added to ethanol–water solution (9:1 vol.%) to hydrolyse it sufficiently. The pH of the solution was adjusted to 3–4 by using acetic acid solution (0.1 mol l^−1^). Then, the hydrophilic silica particles were added into the above solution. The mixture was heated at 60–70°C in a water bath and stirred at 500 r.p.m. for 30 min. Afterwards, the particles were filtered and the silanized product was immersed in deionized water for 24 h, and then washed several times with deionized water to eliminate the influence of physical adsorption. Finally, the silanized silica particles were dried at (103 ± 5)°C for 24 h to obtain a constant weight. The modified silica was labelled as MPTS-2, MPTS-4, MPTS-6 and MPTS-8 for different MPTS loadings.

### 2.3. Silanol content

The silanol (Si─OH) content was determined by acid–base titration of the silica against aqueous sodium hydroxide [30]. About 2 g of silica particles were added to the mixture containing 25 ml of ethanol and 75 ml of 20% (w/w) sodium chloride solution. After that, the silica system was uniformly dispersed by magnetic stirring for 10 min. The pH of the above system was adjusted to 4 with dilute hydrochloric acid (0.1 mol l^−1^). Then, the titration was carried out manually with aqueous sodium hydroxide (0.1 mol l^−1^) to adjust the pH from 4 to 9 and was kept steady for about 20 s. The titre was that volume required to raise the pH from 4 to 9. The silanol numbers of the silica were calculated by the following formula:

$$N(\text{per}\;{\text{nm}}^{2})=\frac{{CVN}_{A}\times 10^{-3}}{Sm},$$

where *N* (per nm^2^) is the number of ─OH groups; *C* (mol l^−1^) is the concentration of the NaOH solution; *V* (ml) is the volume of NaOH solution required to raise the pH from 4 to 9; *N*_A_ is Avogadro's number (6.02 × 10^23^); *S* (nm^2 ^g^−1^) is the specific surface area of silica; and *m* (g) is the weight of silica. The relative content of residual Si─OH on the silica surface was calculated as follows:

$$\text{relative}\;\mathrm{Si}─\mathrm{OH}\;\text{content}\;(\%)=\frac{\mathrm{Si}─\mathrm{OH}\;\text{content}\;\text{in}\;\text{modified}\;\text{silica}}{\mathrm{Si}─\mathrm{OH}\;\text{content}\;\text{in}\;\text{unmodified}\;\text{silica}}\times \text{100 \%}.$$

### 2.4. Pore characterization

The pore size distribution of silica agglomerates was determined by nitrogen adsorption experiments at 25°C on an Autosorb-iQ automatic analyser (Quantachrome, Boynton Beach, FL, USA). The specific surface area (*S*_BET_) was calculated by the BET method based on nitrogen adsorption isotherm data [31]. The total pore volume (*V*_tot_) was evaluated by converting the amount of nitrogen adsorbed at a relative pressure of 0.995 to the volume of liquid adsorbate. The mesopore volume (*V*_meso_) was calculated by the Barrett–Joyner–Halenda method.

### 2.5. Moisture adsorption

The DVS apparatus (IGAsorp; Hiden Isochema Ltd, England) was applied to investigate the moisture adsorption behaviours. The dried samples prepared as above of weight approximately 5 mg were placed in the sample holder, which was connected to a microbalance by a hanging wire and was located in a thermostatically controlled cabinet. A constant flow of nitrogen gas, into which nitrogen containing a preset amount of water vapour was mixed, was passed through the chamber to maintain a given RH. The temperature in the cabinet was kept constant at 25°C, while the RH was increased from 0% to 90% and then decreased back again to 0% at intervals of 5%. The instrument was maintained at each RH value for at least 10 min until the sample moisture content changed by less than 0.002% per min and the value was recorded as the equilibrium moisture content (EMC). Prior to the adsorption process, a drying process at 0% RH was conducted until the sample weight was stable.

### 2.6. Hailwood–Horrobin model

A solid solution-based model, the H-H model [32], was applied to describe the moisture sorption properties of silica. It was extensively used to investigate the sorption behaviour of porous materials containing hydroxyl groups. For simplification, some assumptions were used in the derivation of the H-H model. The adsorbed moisture was assumed to exist in two states, one formed a hydrate with a definite unit of the adsorbent and the second formed a solid solution in the adsorbent. Then, the adsorbent–water system could be assumed to consist of three chemical components; namely, dry adsorbent, hydrated adsorbent and dissolved water, which could be treated as an ideal solution. Here, the three components correspond to dry silica agglomerates, hydrated silica agglomerates with moisture adsorbed on the silica surface via a hydrogen bond, which is surface-bound water (*M*_h_), and dissolved water (*M*_d_)*,* which can be treated as multilayer-adsorbed water.

The sorption equation for this model is expressed as follows:

2.1 $$M=M_{h}+M_{d}=\frac{18}{W}\frac{K_{h}K_{d}h}{1+K_{h}K_{d}h}+\frac{18}{W}\frac{K_{d}h}{1-K_{d}h}$$

and

2.2 $$\frac{h}{M}=A+Bh-Ch^{2},$$

where *M* is the EMC (%); *W* is the molecular weight of the silica substance necessary to bond 1 mol of water (mol mol^−1^); *K*_h_ is the equilibrium constant, defined as the ratio of the activity of silica hydration to the activities of unhydrated silica and dissolved water; *K*_d_ is the equilibrium constant between the dissolved water and the vapour in the atmosphere; and *h* is the RH. By rewriting equations (2.1) and (2.2), the parameters of *A*, *B* and *C*, as well as *K*_h_, *K*_d_ and *W*, can all be derived by plotting *h /M* against *h*; and the calculation can be expressed as follows:

2.3 $$\begin{array}{r} K_{h}=\frac{C}{A{K_{d}}^{2}}, \end{array}$$

2.4 $$\begin{array}{r} K_{d}=\frac{-B/A+{\sqrt{{\left(B/A\right)}^{2}+4C/A}}^{}}{2} \end{array}$$

2.5 $$\begin{array}{r} \;\text{and}\;\;W=\frac{18B{\left(K_{h}+1\right)}^{}}{K_{h}-1}. \end{array}$$

## 3. Results and discussion

### 3.1. Relative silanol content and porous characteristics

**Figure 1.**
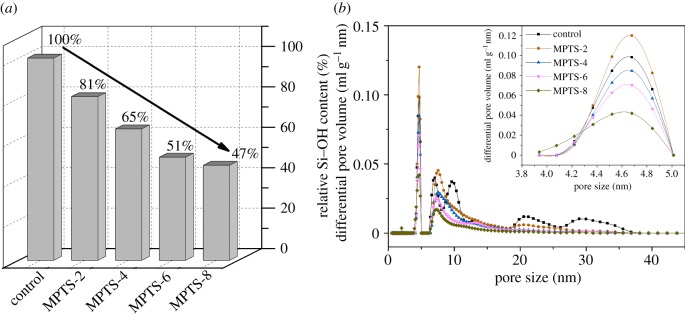
Relative Si─OH content on the silica surface (*a*) and pore size distribution (*b*) of unmodified and MPTS-modified silica agglomerates.

Figure 1*a* shows the relative Si─OH content of unmodified and MPTS-modified silica, which decreased from 100% for the control to 47% for 8% MPTS-modified silica. The relative Si─OH content decreased almost linearly with increasing MPTS concentration. However, above 6%, the decreasing trend was more gradual. This can be attributed to monolayer adsorption between MPTS and silica during the initial process, while, above the critical value, multilayer adsorption of MPTS can occur on the silica surface and introduce extra ─OH groups. The MPTS modification decreased the amount of ─OH groups on the silica surface, which can reduce H-bonding between particles and reduce the agglomeration tendency.

To demonstrate the effect of MPTS on silica agglomerates, figure 1*b* shows the pore size distributions formed by agglomeration of unmodified and MPTS-modified silica, which were obtained by using density functional theory. No micropores (less than 2 nm) were observed in all samples; the dominating structure for nitrogen adsorption was mesopores (2–50 nm). Numerous mesopores were also observed on unmodified silica, indicating the clear aggregation behaviour. The mesopore peaks at 7–11 nm and 26–37 nm in unmodified silica disappeared with the modification by MPTS, which could be attributed to the pore-filling or grafting of MPTS that makes the original mesopores smaller. Therefore, the peaks at 4–5 nm and 6–10 nm changed after MPTS modification. After 2% MPTS modification, the pore volumes within both size ranges were very high, and then with the increase of MPTS concentration, the pore volumes in the mesopore regions (4–5 nm, 6–10 nm, 12–15 nm and 19–25 nm) all showed a decreasing trend, indicating that more and more MPTS filled the pores (grafted on the silica surface) with the increasing concentration. It was suggested that MPTS reacts with the ─OH groups on the silica surface through the formation of hydrogen bonds or covalent bonds, which prevents the formation of the pore structure caused by agglomeration. This reaction was confirmed by previous studies. Rodriguez *et al*. [33] used MPTS to modify a slate surface, which is generally constituted by silicates, and found that MPTS can be adsorbed onto the slate surface by hydrogen bonding. Furthermore, Li *et al*. [22] found the MPTS can be covalently bonded onto a silica surface to improve the surface hydrophobicity of SiC/SiO_2_ powder. Additionally, their study showed that, when the MPTS was overloaded, part of MPTS would participate in multilayer adsorption rather than surface grafting [34].

The parameters of the pores in unmodified and MPTS-modified silica agglomerates, mainly including *S*_BET_, *V*_tot_ and *V*_meso_, are listed in table 1. The results in this table are consistent with those shown in figure 1*b*. The *S*_BET_ gradually decreased with increasing MPTS concentration, whereas the *V*_tot_ and *V*_meso_ decreased abruptly from control to 2% MPTS modification, and thereafter decreased gradually. By calculating the decreasing rate in *V*_tot_, we found that the decrease in *V*_tot_ was significant (68.8%) for MPTS-2 and then became more gradual for MPTS-4 (14.3%), MPTS-6 (12.1%) and MPTS-8 (15.5%). The changing trends between the surface area and volume were due to the different size distributions caused by different degrees of agglomeration due to various degrees of silanization. For unmodified silica agglomerates, the total and mesopore volumes were greater because of the existence of numerous ─OH groups on the silica surface that resulted in more agglomerates. After 2% MPTS modification, some mesopores became smaller due to the grafting (pore-filling) on the silica surface, which resulted in an abrupt decrease in pore volume, but the surface area just decreased gradually because the number of small pores increased, which contributed significantly to the relative increase in specific surface area. With the further increase in MPTS modification, the number of pores decreased, indicating improved dispersity, especially for the smaller mesopores; therefore, the volumes and surface area both decreased gradually.

**Table 1.** Porous characteristics of unmodified and MPTS-modified silica agglomerates. *S*_BET_, specific surface area calculated from the BET method; *V*_tot_, total pore volume; *V*_meso_, mesoporous volume. Decrease in *V*_tot_ was defined as the ratio of the change in *V*_tot_ values for adjacent two samples (e.g. for MPTS-4 = (*V*_tot_ of MPTS-2 − *V*_tot_ of MPTS-4)/ *V*_tot_ of MPTS-2).

label	*S*_BET_ (m^2 ^g^−1^)	*V*_tot_ (cm^3^ g^−1^)	*V*_meso_ (cm^3^ g^−1^)	decrease in *V*_tot_ (%)
control	271.8	2.47	2.44	—
MPTS-2	210.4	0.77	0.63	68.8
MPTS-4	172.1	0.66	0.53	14.3
MPTS-6	145.1	0.58	0.46	12.1
MPTS-8	90.8	0.49	0.37	15.5

### 3.2. Analysis of moisture adsorption data by the Hailwood–Horrobin model

**Figure 2.**
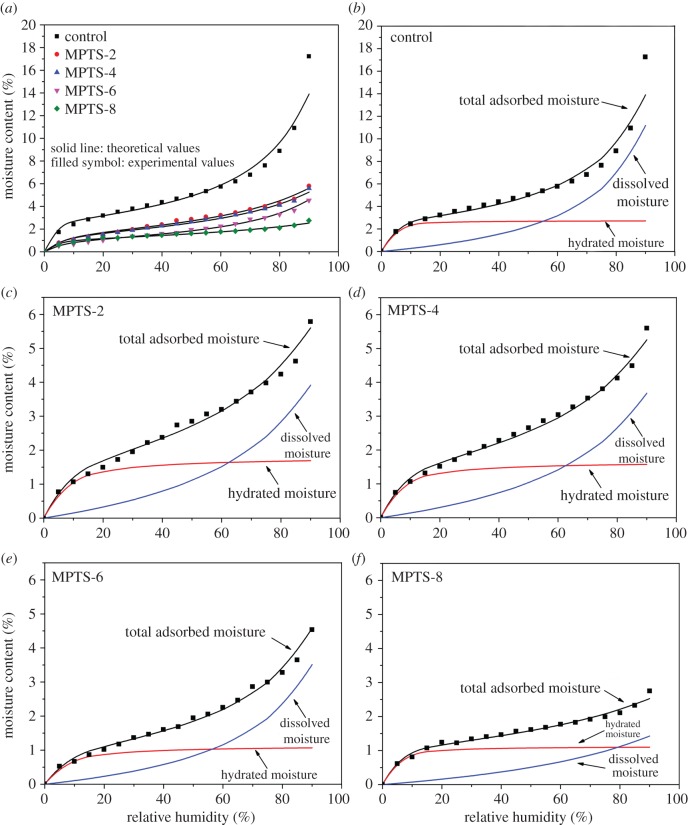
Total adsorbed moisture fits with a best line through the adsorption isotherm data (filled squares) (*a*) and comparison of hydrated water (*M*_h_) and dissolved moisture (*M*_d_) derived from the H-H model (*b*–*f*).

The moisture adsorption data were fitted by the H-H model to obtain the moisture adsorption isotherms (figure 2) shown as type **II** [35], suggesting that the adsorbate–adsorbent interactions were relatively stronger than the adsorbate–adsorbate interactions. This was because the moisture molecules could attract ─OH groups on the silica surface through hydrogen bonds. As shown in figure 2, the moisture content decreased with the increase in MPTS addition. This was because the MPTS is hydrophobic, and could partly replace the ─OH groups on the silica surface via hydrogen bonding or chemical grafting, resulting in the increase in surface hydrophobicity.

The parameters of the H-H model are listed in table 2. The *R^2^* values were all between 0.9386 and 0.9857, suggesting that the regressive equation fitted the experimental data very well. From the change in *K*_h_, it can be seen that the unmodified silica agglomerates were inclined to form hydrated silica during moisture adsorption by showing a high *K*_h_ value of around 83. After MPTS modification from 2% to 6%, the value of *K*_h_ decreased greatly, suggesting a decrease in the formation of hydrated silica. After 8% MPTS modification, this constant increased again to around 65. The reason for this will be discussed later. Also as an indicator of the moisture adsorption state, *W* represents the molecular weight of silica bound to 1 mol of water [32]. With the increase in MPTS concentration, *W* increased gradually with increasing MPTS concentration from 2% to 6% and then changed only slightly, also suggesting a decrease in adsorption sites in silica after MPTS modification up to 6%.

**Table 2.** Hailwood–Horrobin model parameters.

label	*A*	*B*	*C*	*R^2^*	*K*_h_	*K*_d_	*W*
control	0.4845	0.3546	0.0032	0.9685	83.0900	0.0089	6.54
MPTS-2	3.3838	0.5009	0.0040	0.9411	20.4881	0.0076	9.94
MPTS-4	3.0643	0.5523	0.0044	0.9637	24.5832	0.0076	10.78
MPTS-6	5.0321	0.7924	0.0070	0.9386	19.7749	0.0084	15.78
MPTS-8	2.1735	0.8578	0.0054	0.9857	64.6775	0.0062	15.93

**Figure 3.**
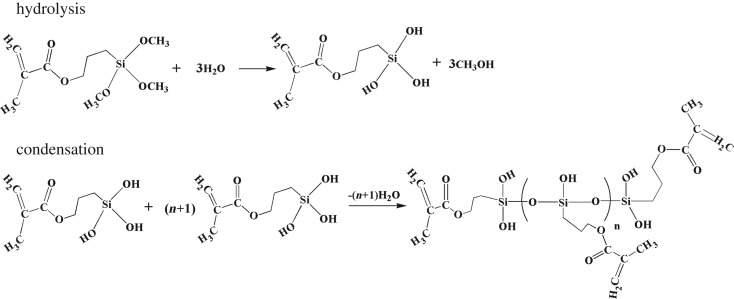
Idealized MPTS self-condensation during silica surface modification.

Using these parameters, the values of *M*_h_ and *M*_d_ can be calculated by equation (2.1). The results for unmodified and MPTS-modified silica agglomerates are presented in figure 2. The *M*_h_ curves are shown as type **I** isotherms [35], which approached saturation after a certain RH. This part of the water was tightly connected with silica by forming hydrated water. Table 3 listed the values of *M*_h_ and *M*_d_ for unmodified and MPTS-modified silica agglomerates at 90% RH. Compared with unmodified silica (2.71%), this saturation point of silanized silica gradually decreased with MPTS concentration within the range from 2% to 6%, and then slightly increased for MPTS-8 (1.10%). This tendency was consistent with the *K*_h_ and *W* results, and can be explained by the reduction of ─OH groups on the silica surface and the self-condensation of MPTS. Namely, when a small amount of MPTS is added, it will occupy the ─OH groups on the silica surface and then reduce the amount of ─OH groups available for moisuture adsorption; but when excessive MPTS is added, these MPTS can undergo self-condensation in the form of multilayers rather than reacting with ─OH groups on the silica surface [33]. This condensation process can produce extra adsorption sites (─OH groups) on the silica surface, as illustrated in figure 3.

**Table 3.** Values of *M*_h_ and *M*_d_ for unmodified and MPTS-modified silica agglomerates at 90% RH.

moisture adsorption types	control (%)	MPTS-2 (%)	MPTS-4 (%)	MPTS-6 (%)	MPTS-8 (%)
*M*_h_	2.71	1.69	1.58	1.07	1.10
*M*_d_	11.18	3.91	3.68	3.51	1.43

Additionally, the *M*_d_ curve shown as type **III** isotherms [35] (figure 2) increased sharply within the whole RH region. The values of *M*_d_ at 90% RH for unmodified and MPTS-modified silica agglomerates are also depicted in table 3. These values decreased with the increase in MPTS concentration, that is, the amount of dissolved water was also influenced by the degree of silanized silica, but this influence included the pore characteristics of the silica agglomerates. As the ─OH groups on the silica surface were firstly occupied by hydrated water, very few or even no ─OH groups were still available for dissolved water adsorption, which made the pore structure become very important in the process of dissolved water adsorption.

### 3.3. Moisture sorption hysteresis

**Figure 4.**
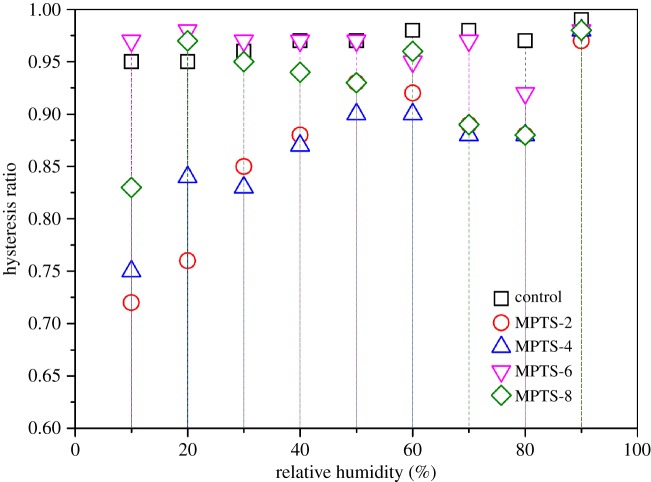
Sorption hysteresis ratio (A/D) distribution of unmodified and MPTS-modified silica agglomerates at different RH conditions (A/D = EMC value of adsorption/ EMC value of desorption at a particular RH).

The EMCs achieved by the adsorption/desorption processes were compared and the hysteresis ratios (A/D) are described in figure 4. It is clear that, at a given RH condition, the A/D values are all lower than 1.0, suggesting that the EMCs from adsorption were lower than those from the desorption process. This phenomenon is referred to as adsorption hysteresis, indicating irreversible mass gain during the adsorption/desorption process. This is partly because the sorption behaviour in mesopores depends not only on the fluid–wall attraction, but also on the attraction interactions between fluid molecules [34]. Especially, water molecules are polar molecules and it is easy for them to form attraction interactions among themselves through hydrogen bonding (40 kJ mol^−1^), and they can strongly interact with the H-bonded ─OH groups on the silica surface, which partly come from the dry state prior to the adsorption [36,37]. Therefore, the moisture adsorption sites in the initial states of adsorption and desorption should be different, that is, the effective adsorption sites for moisture at the beginning of adsorption should be less than those for desorption.

In figure 4, the hysteresis ratio changed with both RH and MPTS concentration. Generally, the hysteresis ratios of unmodified silica agglomerates all changed from 0.95 to 0.99, and increased slightly with increasing RH. After modification with 2% or 4% MPTS, A/D values decreased greatly, especially at low RH conditions. For example, the A/D value for MPTS-2 at 10% RH fell to 0.72. However, with the further increase in MPTS concentration to 6%, the hysteresis ratios increased again and approached the level of unmodified silica. After excessive loading of MPTS at 8%, the hysteresis ratios depended greatly on RH.

**Figure 5.**
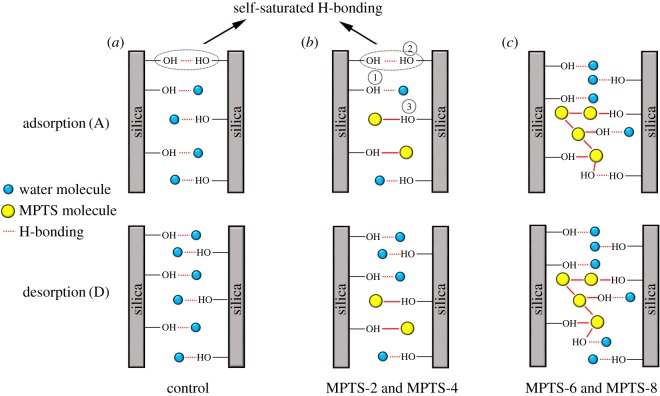
Schematic diagram of the proposed mechanism for the adsorption hysteresis of moisture on the surface of silica agglomerates at a low RH condition in an ideal single cylinder pore.

A schematic diagram was proposed to explain the moisture adsorption hysteresis of unmodified and MPTS-modified silica agglomerates, as shown in figure 5. In this diagram, the ─OH groups on the silica agglomerates’ surface include three types; namely, free ─OH groups (①), H-bonded ─OH groups (②) and MPTS-modified ─OH groups (③). For unmodified silica agglomerates, the adsorption hysteresis can be explained in two ways: one is the reduction in the effective adsorption sites during the adsorption process due to the hydrogen bonding between the hydroxyl groups on the silica surface formed in the dry state (figure 5*a*), and the other is the difficulty of desorption of water molecules from the silica surface because of the narrow mesopores after the adsorption process, which is also referred to as the ‘ink bottle effect' [38,39]. For MPTS-2 and MPTS-4, at RHs below 50%, the ─OH groups on the silica surface were partly replaced by MPTS (figure 5*b*), resulting in a further reduction in the number of moisture adsorption sites. Moreover, the pore size was smaller than that in unmodified silica due to the pore-filling or grafting of MPTS, which led to more difficult desorption of water molecules from silica pores than from the unmodified silica. As a result, A/D showed smaller values than unmodified silica, that is, the hysteresis increased. When 6% and 8% MPTS were added, MPTS condensation (multilayer adsorption) occurred intensively, partly creating extra ─OH groups on the MPTS-modified surface, which might be potential adsorption sites for moisture or to form hydrogen bonds with other ─OH groups (figure 5*c*); also, the pore volume and quantities further decreased (figure 1), all of which resulted in an increase in A/D values compared with MPTS-2 and MPTS-4.

Another interesting finding was that the A/D values of MPTS-modified silica agglomerates increased slightly and were relatively close to those of unmodified silica within the RH range from 50% to 90%. At lower RH levels (less than 50%), hydrated water was predominant in adsorbed water, while at higher RH levels (greater than 50%), dissolved water played a more important role. It is not only the pore structure but also the entire chemical surface of the hydrated silica agglomerates (surface hydroxylation due to hydrated water adsorption) that decreases the pore structure effect. Therefore, at higher RH, the self-saturated hydrogen bonds in the pore can be ignored, and also the dissolved water molecules are easier to desorb from the silica surface than the hydrated water, which resulted in decreasing hysteresis. A similar synergistic effect of pore size and surface chemical characters was also found in the study of cycling water adsorption in micro- and mesoporous silica [40].

## 4. Conclusion

Agglomerated fumed silica particles can form a pore structure, as determined by the nitrogen adsorption experiment. The pore characteristics and moisture sorption behaviour of the agglomerated silica were greatly influenced by MPTS concentration, which resulted in the change of relative Si─OH content on the silica surface. With the increase in MPTS concentration from 2% to 8%, the silanol content decreased from 81% to 47%. Both unmodified and MPTS-modified silica agglomerates were characterized by a 2–50 nm pore structure due to the agglomeration; the pore distribution changed with the MPTS modification at different concentrations. The larger mesopores became smaller or disappeared after MPTS modification, and therefore the number of small mesopores increased after 2% MPTS modification, and then decreased at higher concentrations. Correspondingly, the pore volume decreased abruptly after 2% MPTS, and the BET surface area decreased more gradually with the increase in MPTS concentration.

The H-H model fitted the sorption isotherms very well, and both hydrated water and dissolved water showed decreasing trends with the increase in MPTS concentration, indicating reduced hygroscopicity. Up to 6% MPTS, the amount of ─OH groups showed a decreasing trend, as indicated by reduced *K*_h_ and *W* parameters, which was also confirmed by the silanol content. Adsorption hysteresis appeared for moisture sorption on silanized silica agglomerates, especially in the low RH region and at low MPTS concentration. This could be explained by the synergistic effect of surface silanol content and pore characteristics after modification by different concentrations of MPTS.

## Supplementary Material

Data Availability

## Data accessibility

The datasets supporting this article have been uploaded as the electronic supplementary material.

## References

[1] Murakami K, Iio S, Ikeda Y, Ito H, Tosaka M, Kohjiya S 2003 Effect of silane-coupling agent on natural rubber filled with silica generated *in situ*. J. Mater. Sci. 38, 1447–1455. (doi:10.1023/A:1022908211748)

[2] Oh C, Lee YG, Jon CU, Oh SG 2009 Synthesis and characterization of hollow silica microspheres functionalized with magnetic particles using W/O emulsion method. Colloids Surf. A 337, 208–212. (doi:10.1016/j.colsurfa.2008.12.010)

[3] Castellano M, Conzatti L, Costa G, Falqui L, Turturro A, Valenti B, Negroni F 2005 Surface modification of silica: 1. Thermodynamic aspects and effect on elastomer reinforcement. Polymer 46, 695–703. (doi:10.1016/j.polymer.2004.11.010)

[4] Binks BP, Lumsdon SO 2000 Influence of particle wettability on the type and stability of surfactant-free emulsions. Langmuir 16, 8622–8631. (doi:10.1021/la000189s)

[5] Binks BP, Clint JH 2002 Solid wettability from surface energy components: relevance to Pickering emulsions. Langmuir 18, 1270–1273. (doi:10.1021/la011420k)

[6] Iler RK 1979 *The chemistry of silica: solubility, polymerization, colloid and surface properties, and biochemistry*. New York, NY: Wiley, Inc.

[7] Kiselev AV (ed.). 1957 Surface chemical compounds and their role in adsorption phenomena, pp. 90 and 199. Moscow, Russia: Moscow State University Press.

[8] Legrand AP (ed.). 1998 The surface properties of silicas. London, UK: Wiley.

[9] Björkegren S, Nordstierna L, Törncrona A, Palmqvist A 2017 Hydrophilic and hydrophobic modifications of colloidal silica particles for Pickering emulsions. J. Colloid Interface Sci. 487, 250–257. (doi:10.1016/j.jcis.2016.10.031) 27776283 10.1016/j.jcis.2016.10.031

[10] Carter BO, Wang W, Adams DJ, Cooper AI 2010 Gas storage in ‘dry water’ and ‘dry gel’ clathrates. Langmuir 26, 3186–3193. (doi:10.1021/la903120p) 19938804 10.1021/la903120p

[11] Binks BP, Tyowua AT 2016 Particle-stabilized powdered water-in-oil emulsions. Langmuir 32, 3110–3115. (doi:10.1021/acs.langmuir.6b00140) 27002604 10.1021/acs.langmuir.6b00140

[12] Aristov YI 2013 Challenging offers of material science for adsorption heat transformation: a review. Appl. Therm. Eng. 50, 1610–1618. (doi:10.1016/j.applthermaleng.2011.09.003)

[13] Ng EP, Mintova S 2008 Nanoporous materials with enhanced hydrophilicity and high water sorption capacity. Microporous Mesoporous Mater. 114, 1–26. (doi:10.1016/j.micromeso.2007.12.022)

[14] Furukawa H, Yaghi OM 2009 Storage of hydrogen, methane, and carbon dioxide in highly porous covalent organic frameworks for clean energy applications. J. Am. Chem. Soc. 131, 8875–8883. (doi:10.1021/ja9015765) 19496589 10.1021/ja9015765

[15] Yu J, Le Y, Cheng B 2012 Fabrication and CO_2_ adsorption performance of bimodal porous silica hollow spheres with amine-modified surfaces. RSC Adv. 2, 6784–6791. (doi:10.1039/C2RA21017G)

[16] Radi S, Tighadouini S, Bacquet M, Degoutin S, Janus L, Mabkhot YN 2016 Fabrication and covalent modification of highly chelated hybrid material based on silica-bipyridine framework for efficient adsorption of heavy metals: isotherms, kinetics and thermodynamics studies. RSC Adv. 6, 82 505–82 514. (doi:10.1039/C6RA14349K)

[17] Matsumoto A, Tsutsumi K, Schumacher K, Unger KK 2002 Surface functionalization and stabilization of mesoporous silica spheres by silanization and their adsorption characteristics. Langmuir 18, 4014–4019. (doi:10.1021/la020004c)

[18] Jesionowski T, Ciesielczyk F, Krysztafkiewicz A 2010 Influence of selected alkoxysilanes on dispersive properties and surface chemistry of spherical silica precipitated in emulsion media. Mater. Chem. Phys. 119, 65–74. (doi:10.1016/j.matchemphys.2009.07.034)

[19] Ridaoui H, Donnet JB, Balard H, Kellou H, Hamdi B, Barthel H, Gottschalk-Gaudig T, Legrand AP 2008 Silane modified fumed silicas and their behaviours in water: influence of grafting ratio and temperature. Colloids Surf. A 330, 80–85. (doi:10.1016/j.colsurfa.2008.07.053)

[20] Khalfi A, Papirer E, Balard H, Barthel H, Heinemann MG 1996 Characterization of silylated silicas by inverse gas chromatography: modelization of the poly (dimethylsiloxane) monomer unit/surface interactions using poly (dimethylsiloxane) oligomers as probes. J. Colloid Interface Sci. 184, 586–593. (doi:10.1006/jcis.1996.0655) 8978563 10.1006/jcis.1996.0655

[21] Pantoja M, Díaz-Benito B, Velasco F, Abenojar J, Del-Real JC 2009 Analysis of hydrolysis process of γ-methacryloxypropyltrimethoxysilane and its influence on the formation of silane coatings on 6063 aluminum alloy. Appl. Surf. Sci. 255, 6386–6390. (doi:10.1016/j.apsusc.2009.02.022)

[22] Li C, Feng D, Wang X, Zhu Y 2016 A thermochemical approach to enhance hydrophobicity of SiC/SiO_2_ powder using γ-methacryloxypropyl trimethoxy silane and octylphenol polyoxyethylene ether (7). Appl. Surf. Sci. 360, 45–51. (doi:10.1016/j.apsusc.2015.10.189)

[23] Krysztafkiewicz A, Werner R, Lipska LK, Jesionowski T 2001 Effect of silane coupling agents on properties of precipitated sodium-aluminium silicates. Colloids Surf. A 182, 65–81. (doi:10.1016/S0927-7757(00)00815-3)

[24] Ma PC, Kim JK, Tang BZ 2006 Functionalization of carbon nanotubes using a silane coupling agent. Carbon 44, 3232–3238. (doi:10.1016/j.carbon.2006.06.032)

[25] Yun S, Song QQ, Zhao DM, Qian GM, Li XN, Li W 2012 Study on the inorganic–organic surface modification of potassium titanate whisker. Appl. Surf. Sci. 258, 4444–4448. (doi:10.1016/j.apsusc.2012.01.003)

[26] Hill CAS, Norton AJ, Newman G 2009 The water vapor sorption behavior of natural fibers. J. Appl. Polym. Sci. 112, 1524–1537. (doi:10.1002/app.29725)

[27] Alix S, Philippe E, Bessadok A, Lebrun L, Morvan C, Marais S 2009 Effect of chemical treatments on water sorption and mechanical properties of flax fibres. Bioresour. Technol. 100, 4742–4749. (doi:10.1016/j.biortech.2009.04.067) 19477120 10.1016/j.biortech.2009.04.067

[28] Murrieta-Pazos I, Gaiani C, Galet L, Cuq B, Desobry S, Scher J 2011 Comparative study of particle structure evolution during water sorption: skim and whole milk powders. Colloids Surf. B 87, 1–10. (doi:10.1016/j.colsurfb.2011.05.001) 10.1016/j.colsurfb.2011.05.001 21612896

[29] Pacek AW, Ding P, Utomo AT 2007 Effect of energy density, pH and temperature on de-aggregation in nano-particles/water suspensions in high shear mixer. Powder Technol. 173, 203–210. (doi:10.1016/j.powtec.2007.01.006)

[30] Sears GW 1956 Determination of specific surface area of colloidal silica by titration with sodium hydroxide. Anal. Chem. 28, 1981–1983. (doi:10.1021/ac60120a048)

[31] Brunauer S, Emmett PH, Teller E 1938 Adsorption of gases in multimolecular layers. J. Am. Chem. Soc. 60, 309–319. (doi:10.1021/ja01269a023)

[32] Hailwood AJ, Horrobin S 1946 Absorption of water by polymers: analysis in terms of a simple model. Trans. Faraday Soc. 42, B084-B092. (doi:10.1039/TF946420B084)

[33] Rodriguez MA, Liso MJ, Rubio F, Rubio J, Oteo JL 1999 Study of the reaction of γ-methacryloxypropyltrimethoxysilane (γ-MPS) with slate surfaces. J. Mater. Sci. 34, 3867–3873. (doi:10.1023/A:1004666621479)

[34] Zhang F, Sautter K, Larsen AM, Davis DA, Samha H, Linford MR 2010 Chemical vapor deposition of three aminosilanes on silicon dioxide: surface characterization, stability, effects of silane concentration, and cyanine dye adsorption. Langmuir 26, 14 648–14 654. (doi:10.1021/la102447y) 10.1021/la102447y 20731334

[35] Lowell S, Shields JE, Thomas MA, Thommes M 2012 Characterization of porous solids and powders: surface area, pore size and density, vol. 16 Berlin, Germany: Springer Science & Business Media.

[36] Thommes M 2010 Physical adsorption characterization of nanoporous materials. Chem. Ing. Tech. 82, 1059–1073. (doi:10.1002/cite.201000064)

[37] Hair ML, Hertl W 1969 Adsorption on hydroxylated silica surfaces. J. Phys. Chem. 73, 4269–4276. (doi:10.1021/j100846a039)

[38] Morishige K, Tateishi N 2003 Adsorption hysteresis in ink-bottle pore. J. Chem. Phys. 119, 2301–2306. (doi:10.1063/1.1585014)

[39] McBain JW 1935 An explanation of hysteresis in the hydration and dehydration of gels. J. Am. Chem. Soc. 57, 699–700. (doi:10.1021/ja01307a502)

[40] Saliba S, Ruch P, Volksen W, Magbitang TP, Dubois G, Michel B 2016 Combined influence of pore size distribution and surface hydrophilicity on the water adsorption characteristics of micro- and mesoporous silica. Microporous Mesoporous Mater. 226, 221–228. (doi:10.1016/j.micromeso.2015.12.029)

